# Non-thermal plasma (NTP) treatment of *Trigonella foenum-graecum* L. seeds stimulates the sprout growth and the production of nutraceutical compounds

**DOI:** 10.1186/s12870-023-04710-0

**Published:** 2024-01-06

**Authors:** Iuliana Motrescu, Constantin Lungoci, Mihai Alexandru Ciolan, Gerard Jităreanu

**Affiliations:** 1 Ion Ionescu de la Brad Iasi University of Life Sciences, 3 Sadoveanu Alley, Iasi, 700490 Romania; 2 Research Institute for Agriculture and Environment, 14 Sadoveanu Alley, Iasi, 700490 Romania; 3 Research Center on Advanced Materials and Technologies, Department of Exact and Natural Science, Institute of Interdisciplinary Research, Alexandru Ioan Cuza University of Iasi, Carol I Blvd., No. 11, Iasi, 700506 Romania

**Keywords:** Nutraceuticals, Fenugreek, Plasma agriculture, Non-thermal plasma

## Abstract

The possibility to stimulate the production of some nutraceutical properties of fenugreek (*Trigonella foenum-graecum* L.) sprouts by non-thermal plasma (NTP) processing of the seeds in different conditions was studied. The non-thermal plasma used in this work was a surface dielectric barrier discharge. Two types of processing were performed: direct NTP treatment and NTP with a cover treatment, to simulate the processing of packaged seeds. For all treatments, the effect of pre-soaking of the seeds was studied as well. The analyses of the seeds after processing indicated an increase of the hydrophilicity of their surface for NTP direct treatment as resulted from the water contact angle measurements, which could be due to the strong etching evidenced by scanning electron microscopy imaging. A significant (p < 0.05) increase of the seedling growth, by up to 50%, was found especially for the pre-soaked seeds. These results were correlated with the increase of chlorophyll pigments concentrations, with higher concentrations in the case of NTP direct treatment than for the NTP with cover treatments. Direct NTP treatment for 30 s of dry seeds led to the highest increase of the flavonoid concentration of about three times compared to that obtained for untreated seeds. For the polyphenols and antioxidant activity, NTP with cover treatments proved to be better, with a significant increase, especially for 90 s treatment of the pre-soaked seeds. All the results indicate the possibility of tuning the nutraceutical properties of fenugreek sprouts by NTP treatment.

## Background

The interest in a healthy diet and lifestyle has been increasing lately, especially due to the increased access to information and awareness that the human body is continuously exposed to a multitude of stress factors, from the environment and not only [1]. To overcome these issues, people focus on adapting their dietary choices, one option being the functional foods or superfoods, that not only have a nutritive value but also poses health promotion and disease prevention abilities, which together are referred to as nutraceuticals. The nutritive value comes from increased concentrations of proteins, fibers, and carbohydrates, while the pharmacological actions are usually attributed to secondary metabolites. One important category of nutraceuticals are the sprouts and microgreens, plants in early development stages [2, 3]. They are consumed raw; thus, the benefits are higher than for thermally processed foods or their mature counterparts [4]. Sprouts are great sources of minerals and phytoactive compounds, with many possible uses in a variety of foods for flavor, color, texture, etc. Sprouts of legumes, cereals, and spices have nutrients and compounds that regulate immunity and aid in the prevention of many diseases, deficiencies, and health issues, adding to the nutritional aspect a functional one [2–5]. Not only do they contain such health-benefic compounds, but they also exhibit a high bioavailability [4].

Among the many types of sprouts used as functional foods, is fenugreek (*Trigonella feonum-graecum* L.), a plant originating from middle to southeast Asia. It is one of the oldest medicinal plants, containing high concentrations of soluble fibers, carbohydrates, and amino acids, the seeds and mature plants being mostly used as nutraceuticals [6–12]. Pharmacological actions are attributed to secondary metabolites such as steroids, saponins, polyphenols, flavonoids, alkaloids, lipids, amino acids, carbohydrates, and terpenes. There are many potential health effects reported, some in case studies (e.g. antioxidant, anti-inflammatory, anticarcinogenic, antidiabetic, antianorexic, anthiatherogenic, antihyperlipidemic, galactogogue, antidiabetic, antifungal, hepatoprotective, modulating sexual health activities, lactation aid), some in traditional medicine (e.g. treatment of leg edema, hair tonic, indigestion, lung congestion) [6–12]. There is a lack of knowledge regarding the dose-related effects; fenugreek seed consumption has been also associated with potential consumption issues: in combination with blood-thinners might increase their effect, and some might exhibit allergic reactions, especially people known to be allergic to other plants in the *Fabaceae* family (e.g., peanuts, soybean), with a recommended safety daily consumption limit of 21 g per day per adult (60 kg) [13]. However, these potential negative effects have only been reported for the consumption of seeds. Except for the multiple health benefits, fenugreek is also used in the food industry as an emulsifying agent, food adhesive, food stabilizer, and aid for the development of several food-based products and supplements [12]. Own to its nutraceutical properties, fenugreek is also used as forage crop [14]. It is important to note that the biochemical composition of the plants is larger determined by the environment, despite the fact that the factors responsible for the increased concentrations of some phytochemicals have not been identified [11].

One technology that addresses the increased demand for such superfoods and their sustainable and safe production, is non-thermal plasma (NTP). As a result of electric energy absorption by a gas, ionization occurs and, in some conditions, a plasma discharge is produced. It is a partially ionized gas, for NTP the ionization degree of the gas being less than 10%; it contains ions, electrons, excited atoms and molecules, and radiation, depending on the type of gas and energy used to ignite it. So far NTP has been successfully used to perform different treatments on thermal-sensitive materials such as polymers and biological surfaces, being useful in inactivating the microbial load, destroying cancer cells and promoting tissue oxygenation, and in agricultural and food industry applications [15–22]. NTP was proved to release dormancy in seeds, stimulate the germination and growth of several seed species, legumes and cereals, remove the microbial contamination from their surface, pest control, increase the concentrations of some secondary metabolites, and has other potential benefic effects [23–29]. All these results make NTP processing a suitable green technology, because it is not using any chemicals harmful for the environment and user or that could enter the food chain affecting the consumer.

NTP processing of seeds belonging to the *Fabaceae* family has been reported in the past decade. Mulungu (*Erythrina velutina*) seeds exposed to a plasma jet NTP shown an increase of germination rate believed to be due to the increased water uptake by the seeds after the treatment [24]. A dielectric barrier discharge was employed in the treatment of *Mimosa Caesalpiniafolia* resulting in the increase of seeds wettability, imbibition, and stimulation of germination [30]. The same discharge was also used in the treatment of *Leucaena leucocephala* seeds with similar results of increased wettability and imbibition but without being able to release the dormancy [25]. Improvement of seed germination was reported for black locust (*Robinia pseudoacacia* L.) after processing in NTP [31]. However, the device is not suitable for the treatment of more seeds than one at the same time and seeds had been scarified before NTP processing. Nedved et al. found that both plasma and electromagnetic field treatments can stimulate the growth of red clover plants, as well as the production of some flavonoids in certain conditions [32]. Alfalfa (*Medicago sativa* L.) seeds were processed using an oxygen plasma, the main effect reported being the increase of wettability of the seeds surface [33]. An atmospheric pressure plasma jet produced in argon was used to study some traits such as chlorophyll and antioxidants contents and the activity of some enzyme in fenugreek, as well as genes involved in the synthesis of diosgenin proving that was possible to stimulate the studied biochemical products and increase the stress tolerance of the plants [34]. A drawback of the study was the inability of processing several seeds at a time, reducing the ability to implement it in industry. Several other reports show the ability of NTP to decontaminate the surface of the seeds and seedlings for some other *Fabaceae* family species, stimulation of the germination in some cases, enhancement of growth, and stimulation of some metabolite activities [31].

In the frame of sustainable sprouts production, to reduce the growing time, increase the shelf-life and, most important, stimulate the production of nutraceutical compounds, we have been focusing in NTP treatment of seeds for sprouting. We found both positive and negative effects of seed processing in different conditions, meaning NTP treatments have some threshold up to which germination, growth, etc. are stimulated, and then there is an inhibition of one or all of these processes. As also indicated by the literature, despite not being well studied, NTP processing of the seeds could stimulate the production of secondary metabolites in the resulting plants, thus our idea is to use it for increasing the concentrations of nutraceutical compounds in sprouts. It is believed that these effects are the results of the synergistic action of plasma components, charged and uncharged particles, radiation, electric and magnetic fields, with a strong contribution of reactive oxygen and nitrogen species (RONS) [35]. There is a huge lack of knowledge related to this topic, most reports focusing only on biometric measurements and/or reporting mostly positive effects to the detriment of knowledge and understanding of the interaction mechanisms. For the treatments, we have chosen fenugreek seeds for sprout production, based on the numerous advantages presented in this section. Our aim was to make a detailed study from seed to plant, in different processing conditions, and also using the pre-soaking of the seeds. Our choice was based on studies from a couple of decades ago when it was proven that the imbibition prior to planting stimulated the wheat seeds, especially when the seeds were grown under some stress, similar to our case when NTP treatment acts as an abiotic stressor on the seeds [36]. The device used to produce the NTP is one with a flexible electrode that has proven its ability in inactivating microorganism from the surface of wrapped medical devices [37]. The advantages of such a device are that it produces a mild NTP in open atmospheric air or it can be attached to a package for in-package treatments, it has low operational costs, not making use of expensive equipment, and it can be scaled up for the treatment of larger quantities of seeds.

## Results

**Fig. 1:**
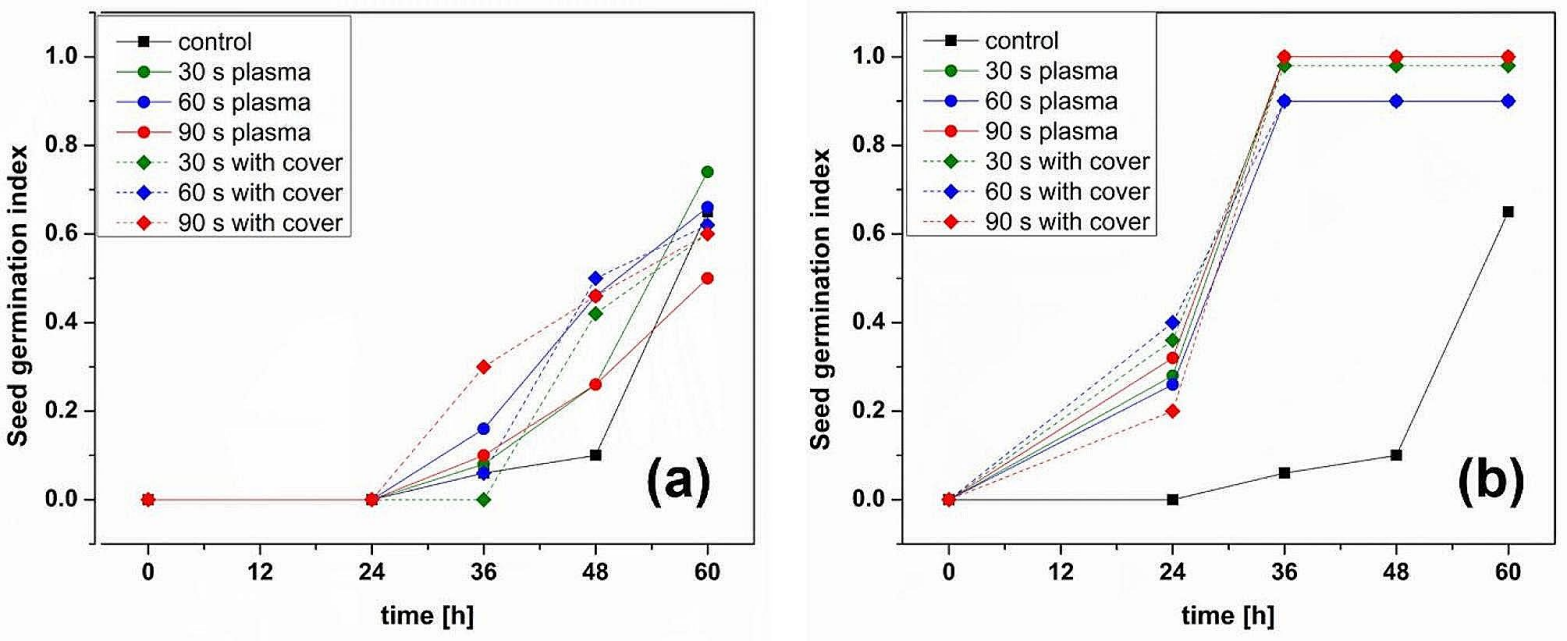
Seed germination index (SGI) vs. time for untreated and treated seeds in different conditions for (**a**) dry seeds, (**b**) 6 h before treatment pre-soaked seeds

Figure 1 shows the seed germination index (SGI) for the seeds treated using direct plasma (continuous lines), plasma with cover (dotted lines), and untreated seeds (in black), for increasing processing time. In almost all the conditions, the germination was better for the treated seeds than for the untreated ones. From Fig. 1 (a) one can see that faster germination was obtained for seeds treated in plasma with cover, especially for 90 s exposure. The situation drastically changes for the pre-soaked seeds (Fig. 1 (b)); seeds start to germinate faster than dry ones and much faster than the NTP untreated ones. After 36 h, all NTP exposed seeds have maximum germination, about one day faster than the dry treated seeds. From the information in Fig. 1 (b) we can also deduct that the treatments with cover up to 60 s are better for germination than only plasma, indicating an important contribution of the reactive species (RONS) that, in this case, are kept near the seeds due to the presence of the cover over the discharge. To better understand the process, we have to look at the other information related to the seeds.

**Fig. 2:**
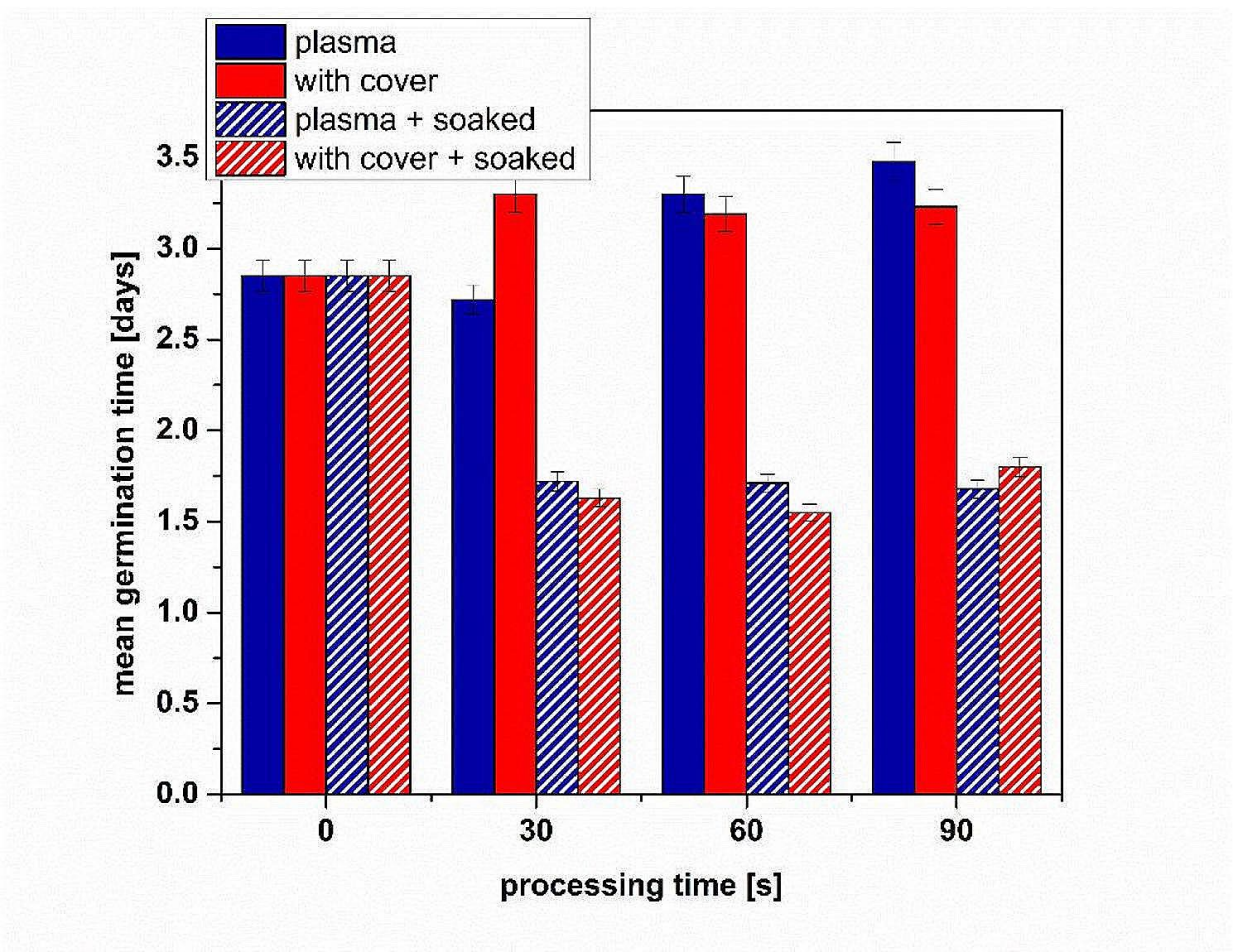
Mean germination time for all the studied experimental conditions

A clearer indication of NTP processing effects for dry and pre-soaked seeds processing can be understood from the evaluation of the mean germination time (MGT). The results in Fig. 2 show the MGT values for all processing conditions, clearly indicating a reduced MGT for the pre-soaked seeds.

**Fig. 3:**
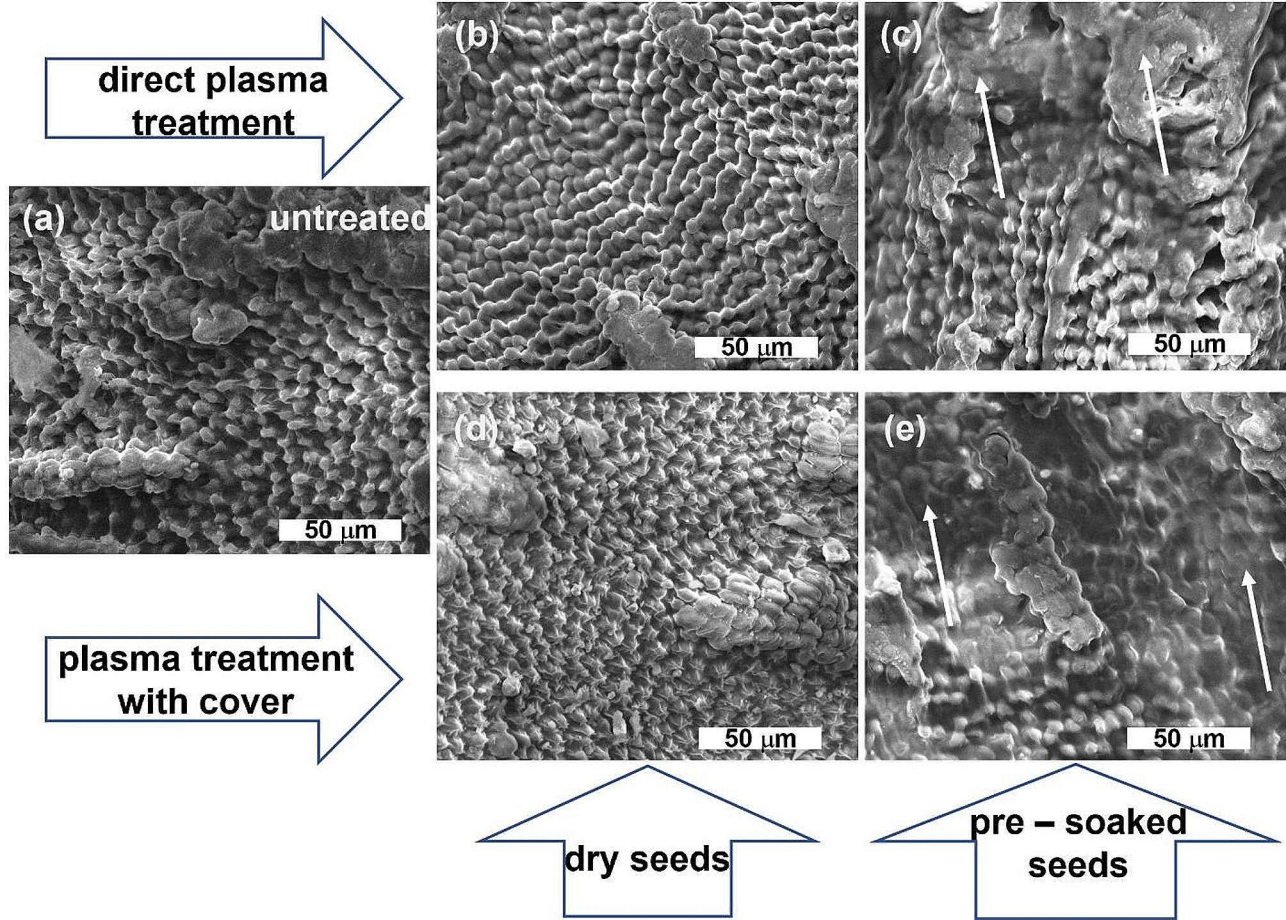
ESEM images of the surface of the seeds in different conditions: (**a**) untreated, (**b**) 90 s dry seeds treated in direct plasma, (**c**) 90 s pre-soaked seeds treated in direct plasma, (**d**) 90 s dry seeds treated in plasma with cover, and (**e**) 90 s pre-soaked seeds treated in plasma with cover. Arrows indicate areas made smoother by strong etching

The morphology of the seeds surfaces changed after NTP treatments in different conditions as can be seen in Fig. 3. Strong surface etching, especially for the pre-soaked seeds and for plasma treatment with cover was detected (Fig. 3 (e)), the surface of the seeds appearing smoother due to this process. For the direct NTP treatment the etching was less extended as indicated by the arrows in Fig. 3 (c). In most studied treatment circumstances and especially after a long treatment (90 s), the appearance of the cells of the aleurone layer was changed. The pre-soaked seeds seem to be more prone to the interaction with the reactive species, radiations, and other particles inside plasma which interact physically and chemically with the outer layer of cells, destroying some of them and removing biological material (Fig. 3 (c) and (e)) and creating a smoother surface.

The results of water contact angle obtained for the dry seeds are shown in Table 1. Direct NTP treatment led to a decrease of the water contact angle, the surface of the seeds becoming more hydrophilic, while direct plasma with cover lead to a negligible decrease of the water contact angle, the behavior being linear with the processing time only for direct plasma treatment. The pre-soaked seeds had already hydrophilic surfaces and was not possible to measure the water contact angle, the drop spreading on the top of the seed.

**Table 1:** Contact angle of the seeds surfaces for the dry seeds in different conditions

	Untreated	30s	60s	90s
NTP	116.75±0.65	118.97±0.43	95.82±0.41	62.03±0.91
NTPwith cover		109.39±1.92	106.8±1.48	115.84±3.00

**Fig. 4:**
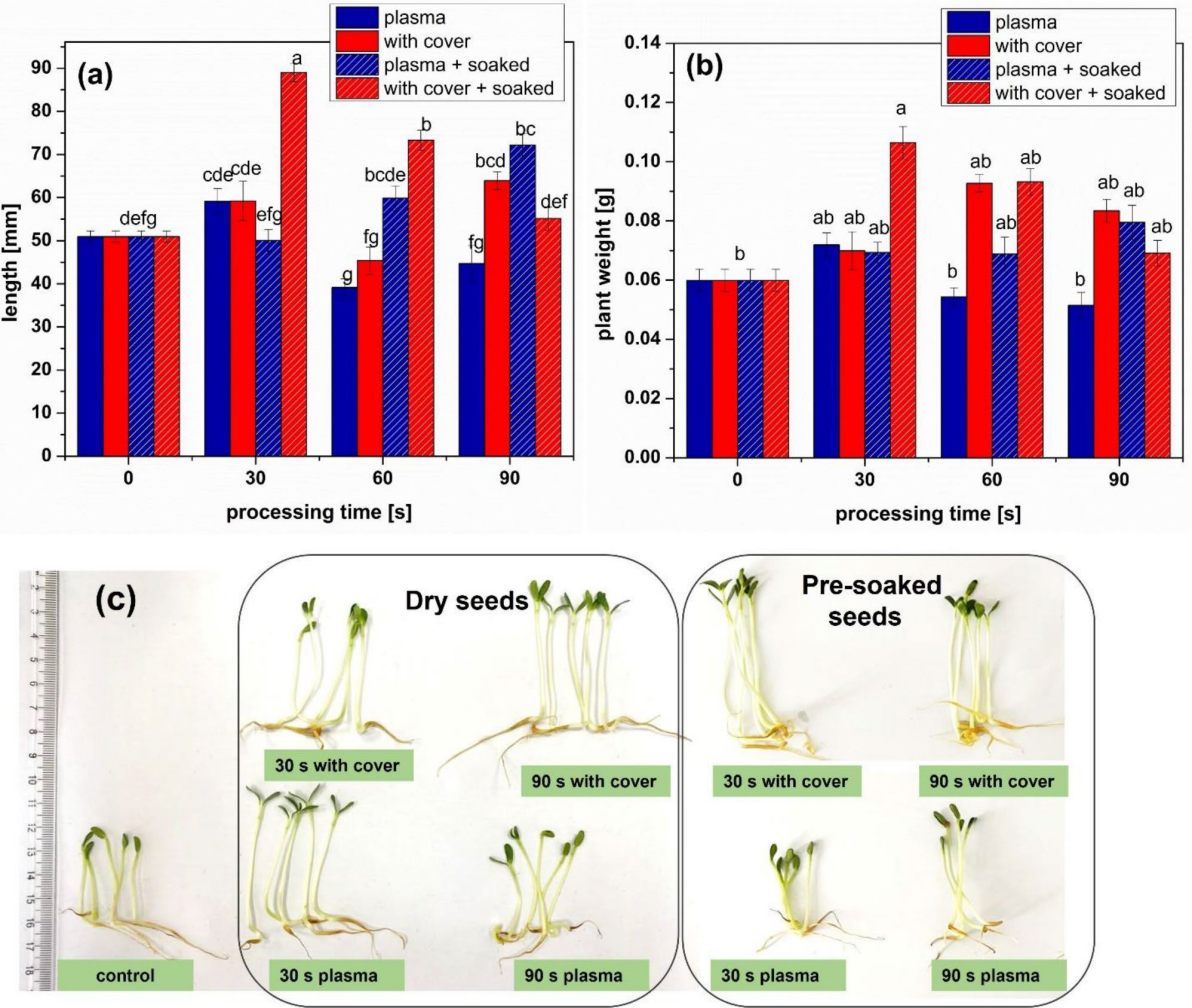
(**a**) Plant length and (**b**) plant weight for sprouts grown from seeds untreated and treated in different conditions as indicated on the figure, versus the processing time (full colors indicate dry seeds, while pattern shaded bars indicate pre-soaked seeds). (Significant differences at 0.05 level are indicated with letters.), and (**c**) sprouts from seeds simultaneously processed in most representative conditions

In Fig. 4 the biometric results of the fenugreek seedlings are presented. In most NTP processing conditions the growth in length and weight of the sprouts was stimulated. For the dry seeds, NTP treatments led to a slight increase of the plant length and mass for 30 s, but inhibited the growth for longer processing times. The same processing conditions but with cover, meaning with enhanced action of the reactive species (RONS), proved to be better than only direct plasma, resulting in sprouts with longer stems. Pre-soaked seeds responded better to all treatments, with increased lengths by up to 41% after plasma treatment and up to almost 75% after treatments with cover indicating that RONS had a very important contribution in the processing. For the pre-soaked seeds, only 30 s with cover treatments led to the strongest stimulation of growth, which shows good premises for implementing such technology into the industrial chain, where a short treatment time means faster processing of a larger quantity of seeds, having economic benefits.

**Fig. 5:**
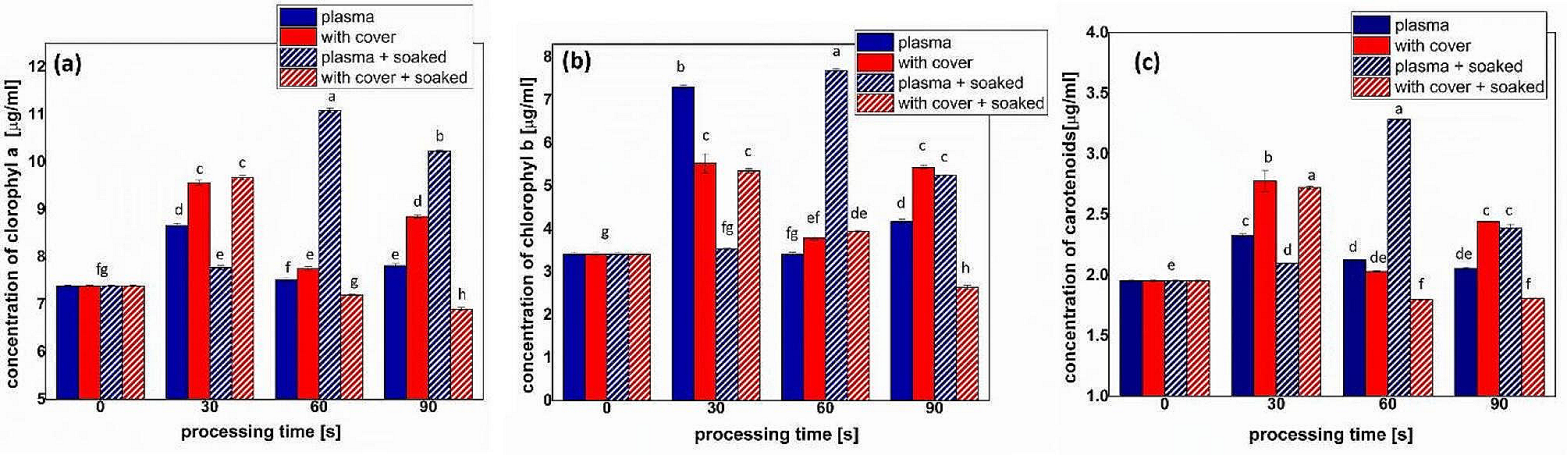
Concentrations of photosynthetic pigments (**a**) chlorophyll *a*, (**b**) chlorophyll *b*, and (**c**) carotenoids, in fenugreek sprouts after seeds treatment in different conditions (full colors indicate dry seeds, while pattern shaded bars indicate pre-soaked seeds). Significant differences at 0.05 level are indicated with letters

Direct NTP treatment of dry seeds resulted in a slight increase of chlorophyll* a* and carotenoid concentrations and a strong increase of chlorophyll *b* for 30 s direct NTP treatment (more than double than for sprouts obtained from untreated seeds) (Fig. 5). The highest chlorophyll *a *content was measured for pre-soaked seeds treated for 60 s in NTP without the cover.

**Fig. 6:**
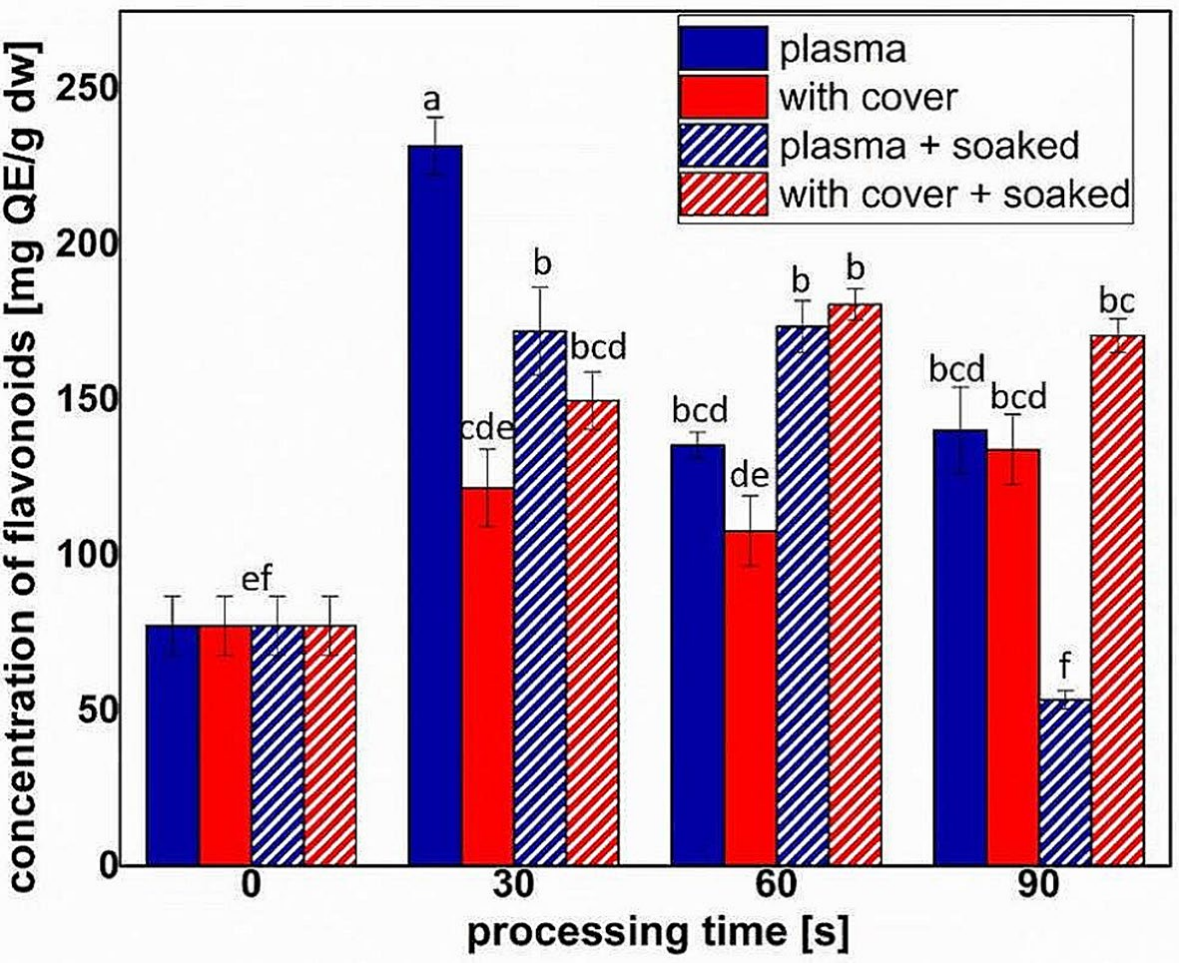
The concentration of flavonoids extracted from sprouts obtained from seeds treated in different conditions vs. treatment time (full colors indicate dry seeds, while pattern shaded bars indicate pre-soaked seeds). Significant differences at 0.05 level are indicated with letters

Figure 6 presents the concentrations of flavonoids for the studied conditions. All the treatments led to concentrations of flavonoids higher than for sprouts obtained from untreated seeds. Only 30 s of direct NTP treatment of dry seeds resulted in a flavonoid content triple than the amount in the control sample, as it can be seen in Fig. 6. For the pre-soaked seeds, the flavonoid concentration almost increased with the treatment time, and was highest for the treatments with cover. Direct treatment of pre-soaked seeds for 90 s led to a smaller concentration than the one measured for control.

**Fig. 7:**
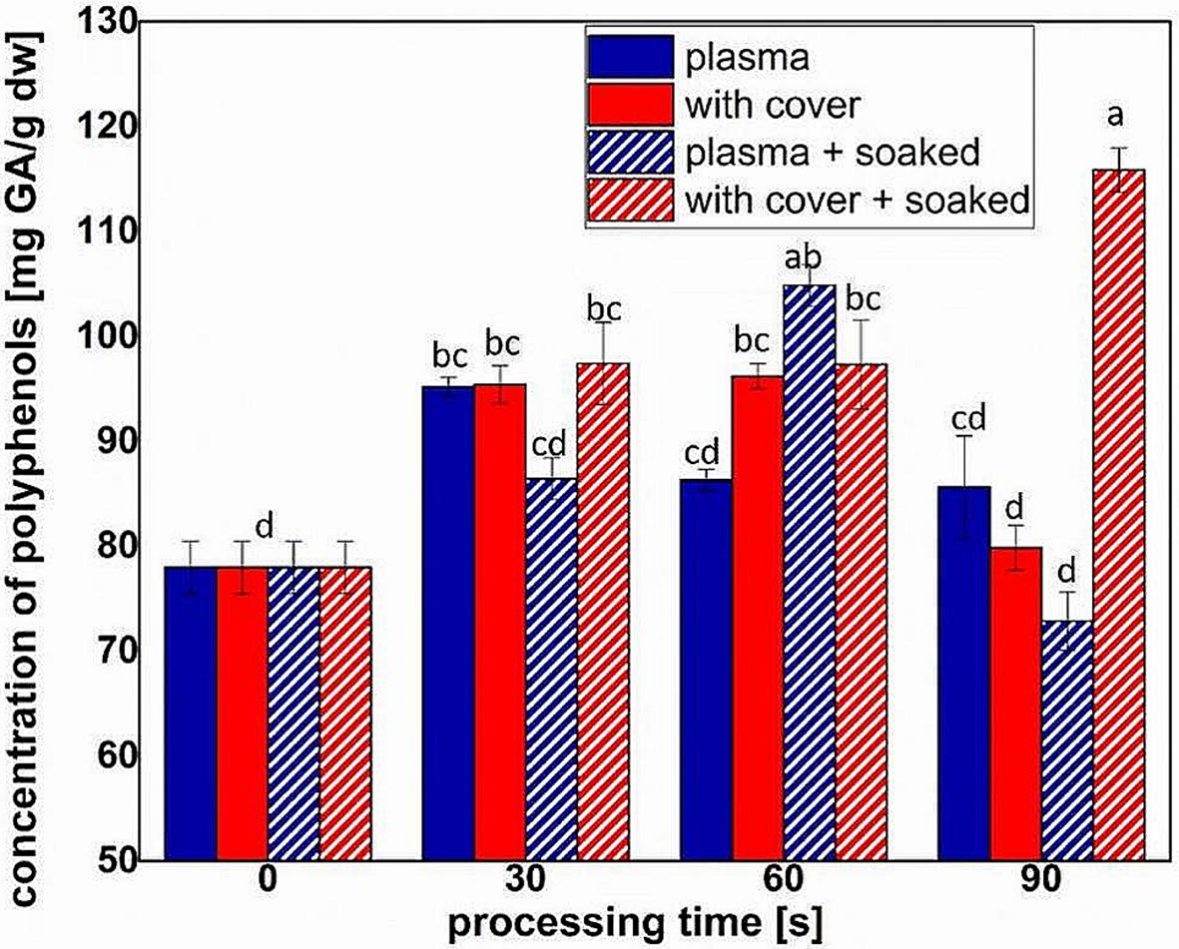
Concentration of polyphenols measured in sprouts obtained for different processing conditions and times (full colors indicate dry seeds, while pattern shaded bars indicate pre-soaked seeds). Significant differences at 0.05 level are indicated with letters

Polyphenols shown a similar behavior, with increased concentrations up to twice those detected for control, and a smaller concentration for 90 s plasma treatment of pre-soaked seeds (Fig. 7). In this case, the concentrations were higher and increased with processing time for pre-soaked seeds treated with cover.

**Fig. 8:**
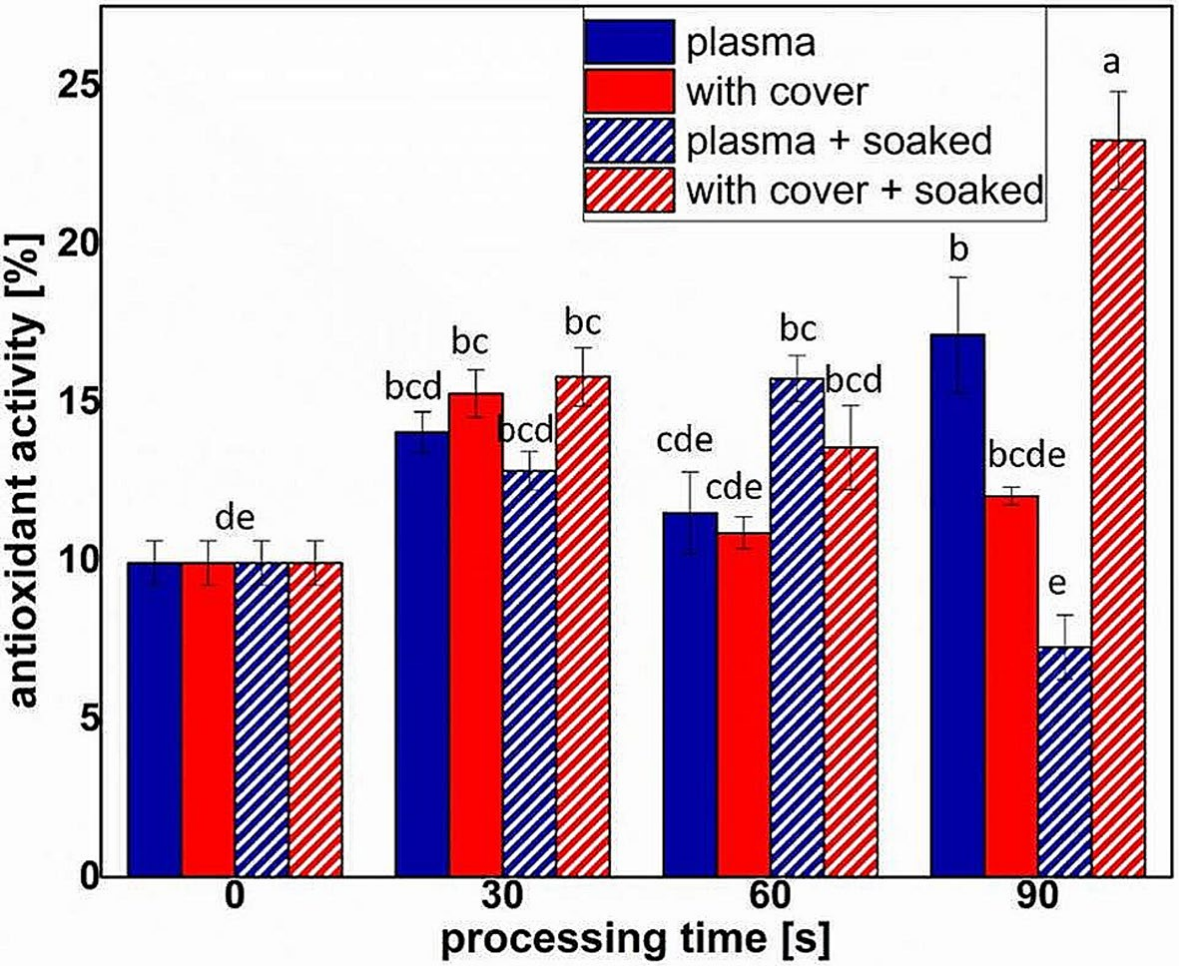
Antioxidant activity evaluated for the sprouts obtained from seeds in different NTP treatment conditions (full colors indicate dry seeds, while pattern shaded bars indicate pre-soaked seeds). Significant differences at 0.05 level are indicated with letters

Figure 8 shows that the highest antioxidant activity was determined for pre-soaked seeds treated in plasma with cover for 90 s, while the smallest for direct plasma of pre-soaked seeds. 30 s of treatment resulted in similar effects for all processing conditions, the strongest changes being observed after 90 s treatment.

The total protein evaluated for sprouts obtained in all conditions had values around 13.2±0.1 mg/g, so this parameter was not affected by any NTP treatment. Despite any major change in the protein content after NTP processing being detected, important information can be drawn from Pearson correlation results presented in Table 2. It was found, as expected, that the total protein was positively correlated with the plant weight and the concentrations of photosynthetic pigments, since photosynthesis leads to the increase of mass and protein production. The protein content shown some small positive correlation with the polyphenols, and, as a result, with the antioxidant activity, and a negative correlation with the concentration of polyphenols.

**Table 2:** Pearson correlation of the experimental results with indication of strong positive correlations in **bold** and strong negative correlations in *italic* (p<0.05)

	Variant	Plant length	Weight	Polyphenols	Flavonoid	Antioxidant activity	Chlorophyll a	Chlorophyll b	Carotenoids	Total protein
Variant	1									
Plant length	-0.156	1								
Weight	-0.201	**0.760**	1							
Polyphenol	0.101	*-0.596*	-0.178	1						
Flavonoids	-0.225	*-0.682*	*-0.520*	**0.610**	1					
Antioxidant activity	0.048	*-0.560*	0.008	**0.817**	0.0570	1				
Chlorophyll a	**0.548**	0.488	0.221	-0.439	*-0.554*	*-0.563*	1			
Chlorophyll b	0.278	0.387	0.156	-0.222	-0.122	-0.370	**0.829**	1		
Carotenoids	0.484	0.358	0.131	-0.348	-0.458	-0.513	**0.932**	**0.822**	1	
Total protein	-0.079	0.356	**0.571**	0.115	-0.138	0.150	0.253	0.315	0.218	1

Correlation is significant at the 0.05 level (2-tailed)

Analyzing Table 2, strong positive correlations were found between flavonoids, polyphenols, antioxidant activity, and between the photosynthetic pigments. Negative correlations between the antioxidant compounds and plant length indicate that NTP processing is complex, the stimulation of plant growth being related to smaller concentrations of nutraceutical compounds. Understanding what happens in all conditions gives great tools for choosing the appropriate treatment for the targeted outcome.

## Discussions

NTP processing of the seeds resulted, as presented in Fig. 3, in strong modification of the seeds surfaces. Etching of the seeds but to a smaller extent was also reported for fenugreek seeds processed in a nonthermal plasma jet discharge in argon [38], with the same NTP device in the case of broccoli seeds [35] as well as in other cases involving NTP processing of other seed species (e.g., cotton, quinoa, rice, wheat) [39–42]. Seed etching happens but the extent of the destruction depends on many parameters, including seed species, sometimes the effects being less visible, as for example in our previous study for cress processed in NTP [35]. Strong morphological changes of the surface of the seeds after plasma treatment have been associated with reduced germination potential and growth of the plants. In the present study we could not find such correlations, probably due to the different structure of fenugreek seed compared to previously studied broccoli and cress.

It is believed that a possible mechanism of germination and growth stimulation as a result of NTP treatment is caused by the increased hydrophilicity of the seed surface [42]. To find possible connections, we measured the water contact angle of the seeds testa. The pre-soaked seeds had hydrophilic surfaces, the drop spreading quickly on top of the surface of the seeds for all processing conditions, including control ones. The increased hydrophilicity must be the results of the surface etching process, as indicated by the micro-imagining of the seeds surfaces. Having in mind the results of germination potential, we do not believe that any change in the surface hydrophilicity due to plasma treatment was strongly connected to the germination of fenugreek seeds. However, the increased hydrophilicity due to pre-soaking of the seeds had a dramatic effect on the germination as we shown by comparing Fig. 1 (a) and Fig. 1 (b). The latter shows a considerable difference between untreated and NTP processed seeds, as well as the GMT values presented in Fig. 2, indicating that, contrary to what was previously believed, the effect of NTP must be related more to other factors than the increased hydrophilicity of the seed surface.

With cover long treatments of pre-soaked seeds inhibited the production of all photosynthetic pigments as presented in Fig. 5. The water absorbed by the seeds during soaking must have had an important role, and because of these results we tend to believe the important contribution of the reactive species that could diffuse inside the seeds during the treatments, facilitated by the water. Chlorophyll b has the role to expand the spectrum of absorption, mostly in the blue light wavelength domain; its increased concentrations were possible adaptation of the plants to NTP stress. The increase of photosynthetic pigments concentrations was also reported for other plants such as medicinal plants under mild salinity stress [43, 44], maize under drought stress [45], or rice under salinity stress [46]. Most existing reports indicate negative effects of abiotic stress factors, but despite the huge amount of knowledge regarding the mechanisms, there is little known about what happens when only the seeds undergo the action of a stressor, not the plant [47]. Some studies found that actually pre-treatment of the seeds using NTP counteracts the action of different abiotic stress factors [46, 48–51]. It seems that the RONS and/or UV radiation produced by NTP induces signaling pathways that promote enzyme activity and increase the concentration of bioactive compounds in plants.

The increase of flavonoid content in almost all the treatments (Fig. 6) shown an important contribution of RONS in the processing outcomes, but also indicates that there were other factors inside plasma that trigger the plant responses. For the polyphenol concentrations (Fig. 7), direct plasma resulted in high concentrations of flavonoids for the dry seeds, while pre-soaked seeds behave better in the treatments with cover. It might be that the water absorbed by the seeds during the soaking facilitated the transport of RONS and triggered processes that resulted in an increase of antioxidant phytochemicals concentrations. The results presented in Figs. 6 and 7 are correlated to those regarding the antioxidant activity, shown in Fig. 8. In none of the cases we did not notice a linear behavior with respect to the processing time, probably also due to the complexity of the analyzed parameters.

Based on the presented results we can say that the metabolic fluxes were readjusted through changes in the photosynthetic rates induced by the interactions between the seeds and plasma components in different circumstances. The result of these changes was reflected in the changed growth pattern, from germination to growth, and can be regarded more as a reorientation of the metabolic response to the action of all plasma components. The mechanism of this short time adaptation is very interesting, especially that the action of the stressor stops before the vegetation phase of the plants when most of the changes are seen. All these are short term changes, and it would also be interesting to study the long-term modifications, if any, by adjusting different processing parameters. The changes that we recorded are adaptations; the increased production of stress metabolites such as antioxidants are stress tolerance mechanisms developed by the plants as a result of plasma processing of the seeds. Studying the behavior under plasma treatment, is clear that under an intense action it is possible to produce negative effects, just as in the case of any other stress factor [35, 40, 41].

The RONS, other charged and uncharged particles, ultraviolet radiation, and electromagnetic field synergically act on the surface of the seed, mostly interacting with the cells in the coat of the seed; RONS might also be penetrating other cell layers [49], being supported by the action of electric field that is known to be facilitating the transport through different cell structures [52]. This penetration could also be enhanced by the presence of water in the case of pre-soaked seeds, as we found that these conditions plus the enhanced concentration of RONS led to an increase of antioxidants and their activity in the resulting plants. The exposure of the seeds to NTP seems to be mostly a eu-stress, as categorized by Lichtenthaler (1996), because after the stress ends, there is a stimulation of different processes, such as germination, growth, production of different phytochemicals, a positive action for the development of plants [53]. Higher discharge voltages that result in higher concentrations of reactive species and more energy dissipated on the seeds, long treatment times, and other such factors can have a negative influence, producing damaging effects on the resulting plants, as reported in the literature [35, 54–57]. Unfortunately, in the case of plasma it is hard to define a dose-effect relationship, due to the variety of discharge configurations, different concentrations and variety of plasma species, including RONS, and so on. Not to forget, as previously mentioned, about the different responses between plant species. Thus, it is also hard to define a threshold.

## Conclusions

In this work we focused on the possibility of increasing the nutraceutical properties of fenugreek used as sprouts by NTP plasma processing of dried and pre-soaked seeds. Germination and biometric measurements indicated that the NTP treatment stimulates the germination of the seeds, especially in the case of pre-soaked seeds, the full germination potential being reached faster than for untreated seeds, and NTP processing with cover, meaning with increased concentrations of RONS lead to larger plants, especially for short treatments and pre-soaked seeds, indicating the important contribution of both reactive species and adsorbed water onto the seeds before the treatments. Direct plasma treatment of pre-soaked seeds also led to an increase of all photosynthetic pigments, mostly after 60 s of seed processing. The total flavonoid concentration, polyphenol concentration, and antioxidant activity increased in most treatment circumstances. These changes are the effects of RONS, electric field and other plasma components, being believed that the water adsorbed by the seeds during pre-soaking facilitates the penetration of RONS and initiating stress-responses in the plants. All these results show the possibility to tune NTP plasma processing of the seeds such as to increase some nutraceutical properties of the resulting pants. More studies are needed in order to separate the contribution of different plasma components on the seeds and figure out in detail the mechanisms for a better control of the outcome.

## Methods

### Seed preparation

Organic fenugreek (*Trigonella foenum-graecum* L.) seeds were commercially procured (Rapunzel Naturkost GmbH, Legau, Germany). Seeds were treated as they were or soaked before the treatments. For the pre-soaking, the seeds were left in pure water for 5 min, then taken out and left to dry in open air on absorbing paper. The soaking took place 6 h before the NTP treatments, such as the seeds would have had time to dry in open air. For each condition untreated samples were kept, germinated, and analyzed as control samples (both dry and soaked seeds).

### Evaluation of the germination

20 seeds from each processing conditions and untreated were put in Petri dishes covered with paper, with 3 ml of pure water and incubated in a controlled environment with a light regime of 12 h light/12 h dark at about 22 °C and a relative humidity of between 45 and 50% in controlled growth chambers [56]. The experiment was performed in triplicate. After incubation, the germination was recoded at 24, 36, 48, and 60 h. The seed germination index (SGI) was evaluated as the ratio between the number of germinated seeds and total number of seeds put to germination; the mean germination time (MGT) was estimated as the ratio between the product of number of germinated seeds each day and the day since sowing and the total number of germinated seeds [58, 59].

### NTP device and processing conditions

**Fig. 9:**
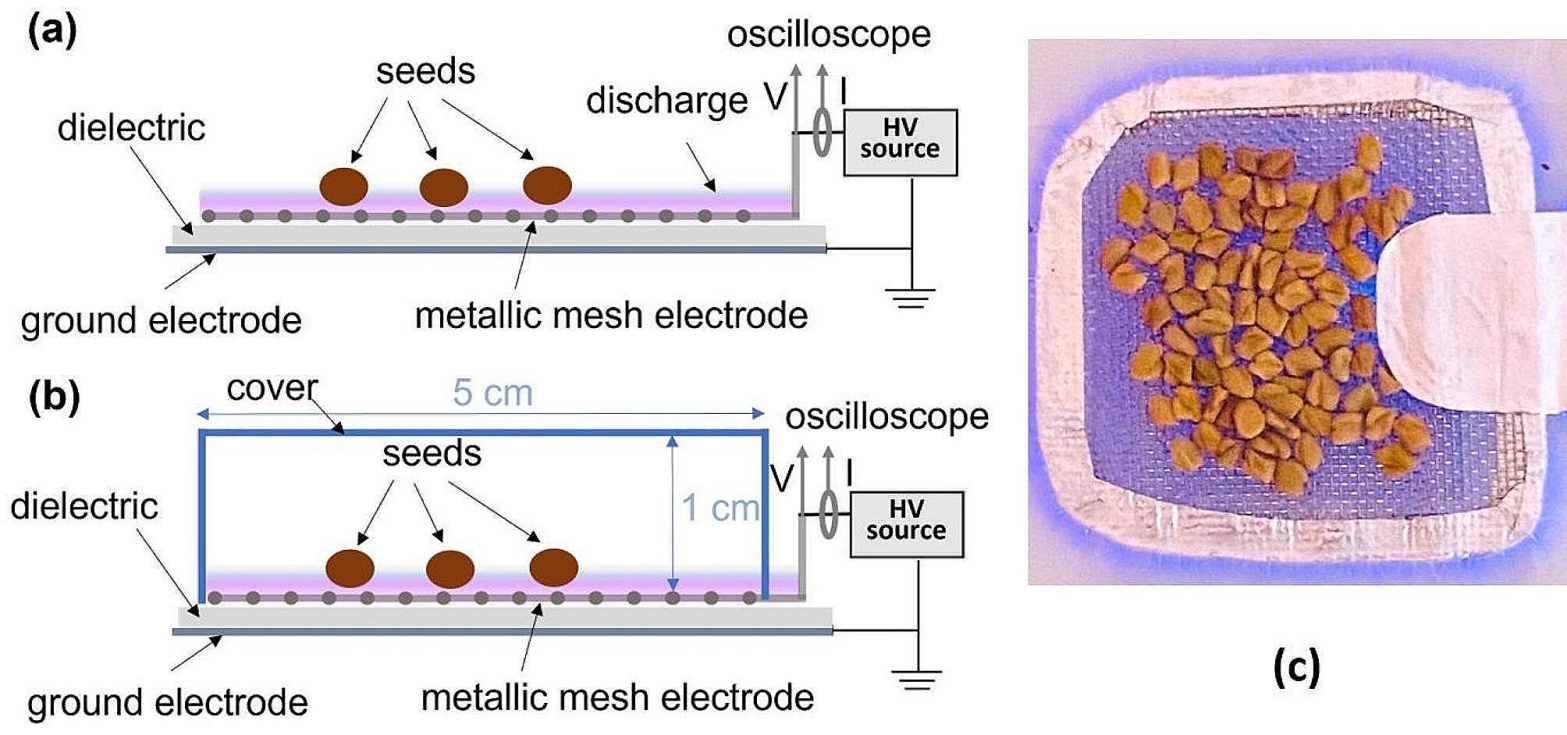
Experimental device (**a**) for NTP direct treatment, (**b**) for NTP with cover treatments, and (**c**) appearance of the discharge electrode with some seeds during NTP direct treatment

The NTP device consisted of a dielectric sheet with two plane electrodes on both sides: a grid electrode serves as driving electrode, while aluminum tape is used as ground electrode (Fig. 9). A 10 kHz signal at 11 kV peak-to-peak was applied between the electrodes to produce a surface dielectric barrier discharge [35]. We chose these conditions based on our previous results because at this voltage we got a mild treatment of broccoli seeds with stimulated growth, while for larger voltages the number of damages produced increased considerably. The device can be attached to a closed package such as that in-package treatments can be performed. The electric and optical emission diagnosis of this plasma was presented for other seed treatments [35]. The treatments were performed for 30, 60, and 90 s. In our previous study we got that longer treatments are damaging on one hand, and longer processing is not desirable in industry on the other hand.

For the direct treatments, seeds were placed to cover the driving electrode in one layer as shown schematically in Fig. 9 (a) and a real photo of the seeds during the treatment in Fig. 8(c); in the case of fenugreek that means about 50 seeds. To simulate the in-package treatments, a cover was placed on top of the seeds put over the driving electrode, as schematically indicated in Fig. 9(b). In this situation, the reactive species (RONS) produced in the air remain in the vicinity of the seeds; otherwise, they would diffuse away from the electrode. During the treatments, the discharge current and voltage were monitored using a current probe (Pearson, 4100, Palo Alto, CA, USA), and an HV probe (Tektronix, P6015A, Beaverton, OR, USA), respectively, on an oscilloscope (Rigol DS2072A, Rigol Technologies, Inc., Beijing, China).

### Analyses of the seeds

The determination of the wettability and morphology of the surface of the seeds was performed by measuring the water contact angle of the seeds surfaces using the drop method. A 1 µL pure water drop was placed on the surface of a seed, then a picture was taken and the water contact angle was determined using the drop snake plugin of imageJ software [60].

The surface of the seeds prior and after the treatments was imaged using Environmental Scanning Electron Microscopy (ESEM) with Quanta 450 electron microscope from FEI (Thermo Fisher Scientific, Hillsboro, OR, USA) in low vacuum mode. The seeds were put on aluminum stubs using double sided carbon tape. The device does not request covering the samples with a metallic layer, so they were analyzed as they were, using 15 kV accelerated electron beam, at different magnifications.

### Biometric measurements

In the 8th day after the treatments, biometrical and mass measurements were performed. The length of the sprouts was measured with a normal ruler, and the mass was determined with a 5 digits analytical balance (Shimadzu AUW220D, Shimadzu Corporation, Kyoto, Japan).

### Spectrophotometric analyses

After performing the biometric measurements, extracts were made from the sprouts for different spectrophotometric analyses: chlorophyll contents, flavonoids and polyphenols contents, antioxidant activity, and total protein.

For evaluating the chlorophyll pigments, 5 g of fresh leaves were grinded, then filtered using 96% ethanol. The absorption of the extract between 200 and 900 nm was determined using a spectrophotometer (Specord 210 Plus, Analytikjena, Jena, Germany), and the absorbances at 470, 649, and 665 nm against a blank sample, were used to calculate the concentrations of chlorophyll *a*, chlorophyll *b*, and carotenoids according to Lichtenthaler (1983) formulas adapted by Wellburn (1994) [61, 62].

0.25 mL of plant extract was mixed with 5% NaNO_2_ and 10% AlCl_3_ in basic pH (NaOH 1 M); the absorbance at 510 nm was used to determine the flavonoid content using a calibration curve made for quercetin (r^2^ = 0.9983) [63].

For the measurements of total phenol content, ethanol extracts were prepared in the same way as for the chlorophyll pigments measurements only using the whole plant, then sonicated for 15 min, incubated for 24 h at 4 °C, and finally filtered. The total phenol concentration was determined using Folin-Ciocalteu reactive; the concentrations expressed in GA/g dw were evaluated from the absorbance at 760 nm after a calibration curve was obtained for gallic acid (r^2^ = 0.9947) [64].

The total protein was determined using the Bradford method with bovine serum albumin as standard (r^2^ = 0.9911) [65]. The measurements were performed after extraction from leaves using Bradford reagent.

### Statistical analysis

The data was analyzed using one-way variance test (ANOVA) (IBM SPSS v14) to determine the significant differences between the treatments and for all the values of the measured parameters. When the results were significant (p < 0.05), Tukey’s multiple comparison test was employed for establishing which means from a set are different from the rest. All the results are presented as averages with standard deviations, as well as indication of significant differences determined by Tukey’s test and indicated by letters on each chart. Correlations between the analyzed parameters were studied performing Pearson correlation test performed with the same software.

## Data Availability

Data can be found here Motrescu, Iuliana (2023), “Fenugreek sprouts”, Mendeley Data, V1, 10.17632/rwfs6wkp3z.1.

## Abbreviations

NTP: non-thermal plasma
RONS: reactive oxygen and nitrogen species
UV: ultraviolet
